# Rapid development of entity-based data models for bioinformatics with persistence object-oriented design and structured interfaces

**DOI:** 10.1186/s13040-017-0130-z

**Published:** 2017-03-11

**Authors:** Elishai Ezra Tsur

**Affiliations:** Neuro-Biomorphic Engineering lab, Faculty of Engineering, Jerusalem College of Technology, Jerusalem, Israel

**Keywords:** Specialized databases, Object-relational databases, EclipseLink, Apache Derby, Object oriented programming

## Abstract

Databases are imperative for research in bioinformatics and computational biology. Current challenges in database design include data heterogeneity and context-dependent interconnections between data entities. These challenges drove the development of unified data interfaces and specialized databases. The curation of specialized databases is an ever-growing challenge due to the introduction of new data sources and the emergence of new relational connections between established datasets. Here, an open-source framework for the curation of specialized databases is proposed. The framework supports user-designed models of data encapsulation, objects persistency and structured interfaces to local and external data sources such as MalaCards, Biomodels and the National Centre for Biotechnology Information (NCBI) databases. The proposed framework was implemented using Java as the development environment, EclipseLink as the data persistency agent and Apache Derby as the database manager. Syntactic analysis was based on J3D, jsoup, Apache Commons and w3c.dom open libraries. Finally, a construction of a specialized database for aneurysms associated vascular diseases is demonstrated. This database contains 3-dimensional geometries of aneurysms, patient’s clinical information, articles, biological models, related diseases and our recently published model of aneurysms’ risk of rapture. Framework is available in: http://nbel-lab.com.

**Electronic supplementary material:**

The online version of this article (doi:10.1186/s13040-017-0130-z) contains supplementary material, which is available to authorized users.

## Background

In the last few decades the intersection of computer science and biology has evolved to the point at which answers to fundamental biological questions have emerged [1]. Some of the most important cross-talks between biology and computer science lie within the data-intensive nature of modern biology [2]. It is currently evident that fields such as computational biology and bioinformatics are practically fueled by the increasing computational resources available and the development of software encapsulation and abstraction layers [3]. An important corner stone of the computer-science/biology interface is object-centered reductionism where relations between discrete biological entities such as DNA, protein and RNA are investigated [1]. Data regarding biological entities is stored in databases, which have become the most important corner stone for research in computational biology and bioinformatics.

Biological database designers currently face two main challenges: data heterogeneity and the emergence of new relational connections between data entities. Today, biological data is not limited to sequential information, which is typically stored in primary databases such as NCBI’s Nucleotide and Protein data sets. Biological data also encompass graphs [4], statistical models [5], geometric information [6], vector fields [7], patterns [8], images [9], computational models [10] and others. A recent important advance regarding data heterogeneity was developed by Allan and colleagues, who have developed OMERO, an open source software platform which uses a server-based middleware application to provide a unified interface for images, matrices and tables [9]. However, while OMERO provides a unified interface for file types, it is currently limited to microscopy images. Another important effort is the development of the Semantic Web Languages (SWL), which promotes web-based standardization of data formats by utilizing eXtensible Markup Language (XML) and Resource Description Framework (RDF). SWL have been implemented by many biological portals such as MGED Ontology, which provides terms for annotating microarray experiments, BioPAX, which provides an exchange format for biological pathway data, and Gene Ontology (GO), which describes biological processes, molecular functions and cellular components of gene products [11].

The management of relational connections between biological data entities is a great challenge due to the variety of contexts in which data can be related. The vast spectrum of possible relations between biological entities drove the momentum for the curation of specialized databases. Specialized databases include organism-centered datasets such as Flybase (Drosophila) [12], WormBase (Nematode) [13], AceDB (C. elegans) [14], and TAIR (Arabidopsis) [15]; biological pathways such as MetaCyc and Biocyc [16]; and diseases such as NCBI’s OMIM database. Today, specialized databases are often curated to serve consortiums and single laboratories that define their own data relations architecture with their own data format. Specialized database curation is an ever-growing need since new data sources are constantly evolving due to rapidly advancing biological research: new experimental techniques produce types of data greater in both variety and number, requiring database structures to change accordingly. Moreover, most specialized databases contain both new data and data that were derived from established datasets. This hybrid approach of the new and the old presents a major challenge to specialized database designers, which should query, acquire and parse data from existing databases, as well as integrate it into their own database architecture.

The relational model, in which sets of tables are used to organize data, was first introduced by Edgar Codd in 1970 and is currently the most widely used model for data representation [17]. Although relational DataBase Management Systems (DBMS) have often been used for biological data management, they are in many ways inadequate. One of the main reasons for this inadequacy is that a relational data model cannot accurately encapsulate important biological data structures such as pedigrees, taxonomies, maps, networks, cascade processes, etc. Moreover, while application development techniques and programming languages have evolved significantly over the past decades, the relational database technology has remained relatively unchanged, frequently causing discrepancies between the object-oriented model used by many modern applications and the relational model [18]. However, while relational databases may be inconvenient to consume by modern programming languages, they are still the main choice for many applications due to their maturity and reliability. One of the main alternatives to relational data model is object-based representation of information, in which entities are defined with a set of properties and connected as attributes. Object-Oriented DataBase Management System (OODBMS), popularized as NoSQL, allows the encapsulation of internal details of the data associated with the heterogeneity of the underlying data sources, extending object-oriented programming with a data persistency. Data persistency allow objects to be created, stored and used directly with no need to explicitly serialize objects to or from a data base. Examples to OODBMS include hbase and DocDb. Importantly, a technique called object-relational mapping (ORM) allows the user to use SQL-like queries while dealing directly with objects, creating a hybrid of both object and relational approaches.

Here, an ORM-based open-source platform is proposed. The platform provides an efficient way for researchers to curate their own database using their own model of encapsulation, while providing direct access to external biological databases such as the National Center for Biotechnology Information (NCBI) databases, MalaCards, Biomodels and others using structured interfaces.

## Implementation

### Framework

**Fig. 1:**
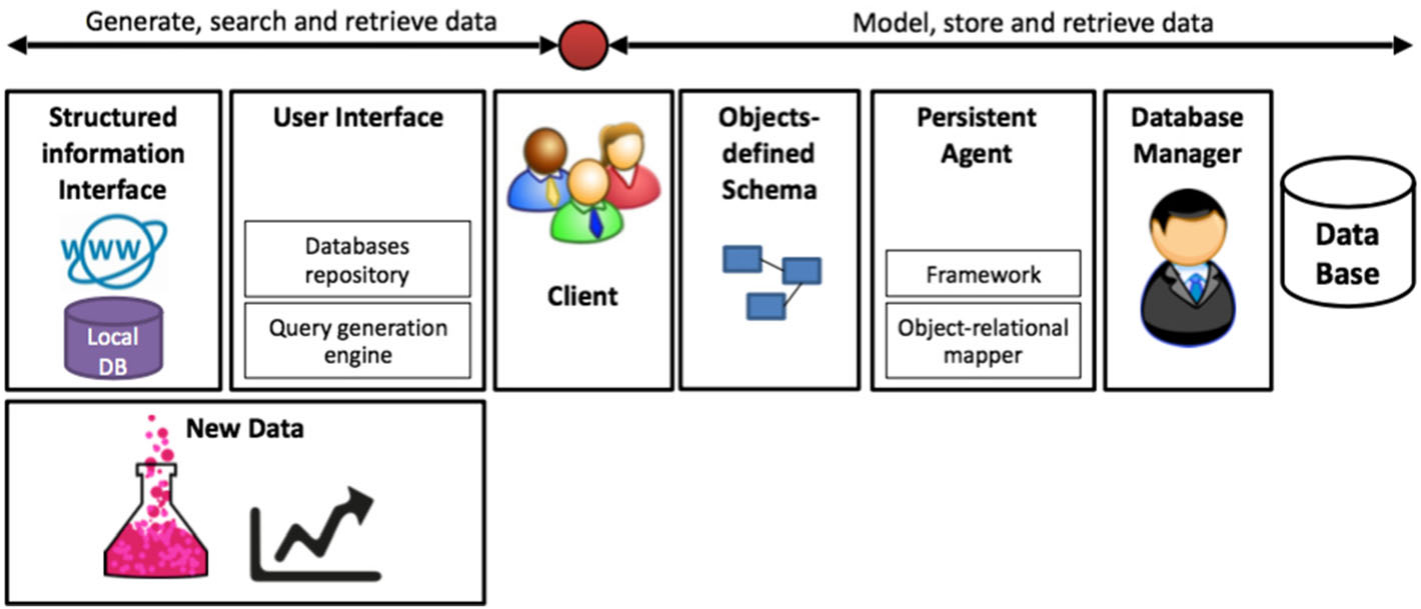
Framework schematics. Our framework is composed of structured information interfaces, used to search and fetch information from local and online repositories, incorporation of user generated new data, query generation interface and data source selection, a persistent agent which persists user-defined object schema to memory and finally, a database manager. This framework supports generation, search and retrieval of data, as well as modeling, storing and searching persisted schema

Here, a simple framework for rapid development of specialized databases is proposed. This framework integrates several database technologies into a unified platform which allows the user to search and retrieve data from existing sources, incorporate established data with newly discovered information in a single data model, define object-based data architecture and persist it to memory for future inquiries. The framework consists of three main data streams: existing data sources to the user (search and retrieve data), new information sources such as experiments and data analytics to the user (generate data), as well as the user herself to a self-curated specialized data-base (model, store and retrieve data) (Fig. 1). The platform enables the user to search and retrieve data from local databases using structured information interfaces, and from online databases using structured URL interfaces. The user interface consists of a database repository which defines the differently structured accesses to existing resources, and a query generation engine which provides advanced searching mechanisms such as field-oriented search and the use of logical relations among search terms. The user designs her own data architecture using Object Oriented Programming (OOP), within which she encapsulates her new data and the database-derived information to class-defined entities, an action which forms the database schema. Entities are persisted to memory with a Persistent Agent (PA). The PA uses an Object Relational Mapper to map the object-based schema to a set of inter-connected tables which resemble a typical relational database. This will allow the user to query her database with SQL-like queries. The relational model is transferred to a database manager that stores the data in memory.

This framework can be implemented in various ways using different programming languages and libraries providers. For example, Southern and colleagues developed a Java API, which maps entities to NCBI’s PubChem schema and provides wrapper functions for calling NCBI eUtilities and PubChem web services [19]. Another implementation is the BioPython project, which provides modules to access NCBI’s databases from within Python [20]. Objects persistence can be achieved both in Python using its standard library, which support a family of hash-based file formats and objects serialization, and in JAVA using Java Persistence API (JPA). Most JPA persistence providers offer the option to automatically create the database schema based on metadata. Popular implementations of JPA are Hibernate, EclipseLink and Apache OpenJPA. Common database managers include Apache Derby, Firebird, SQLite, and HSQL.

### Software description

**Fig. 2:**
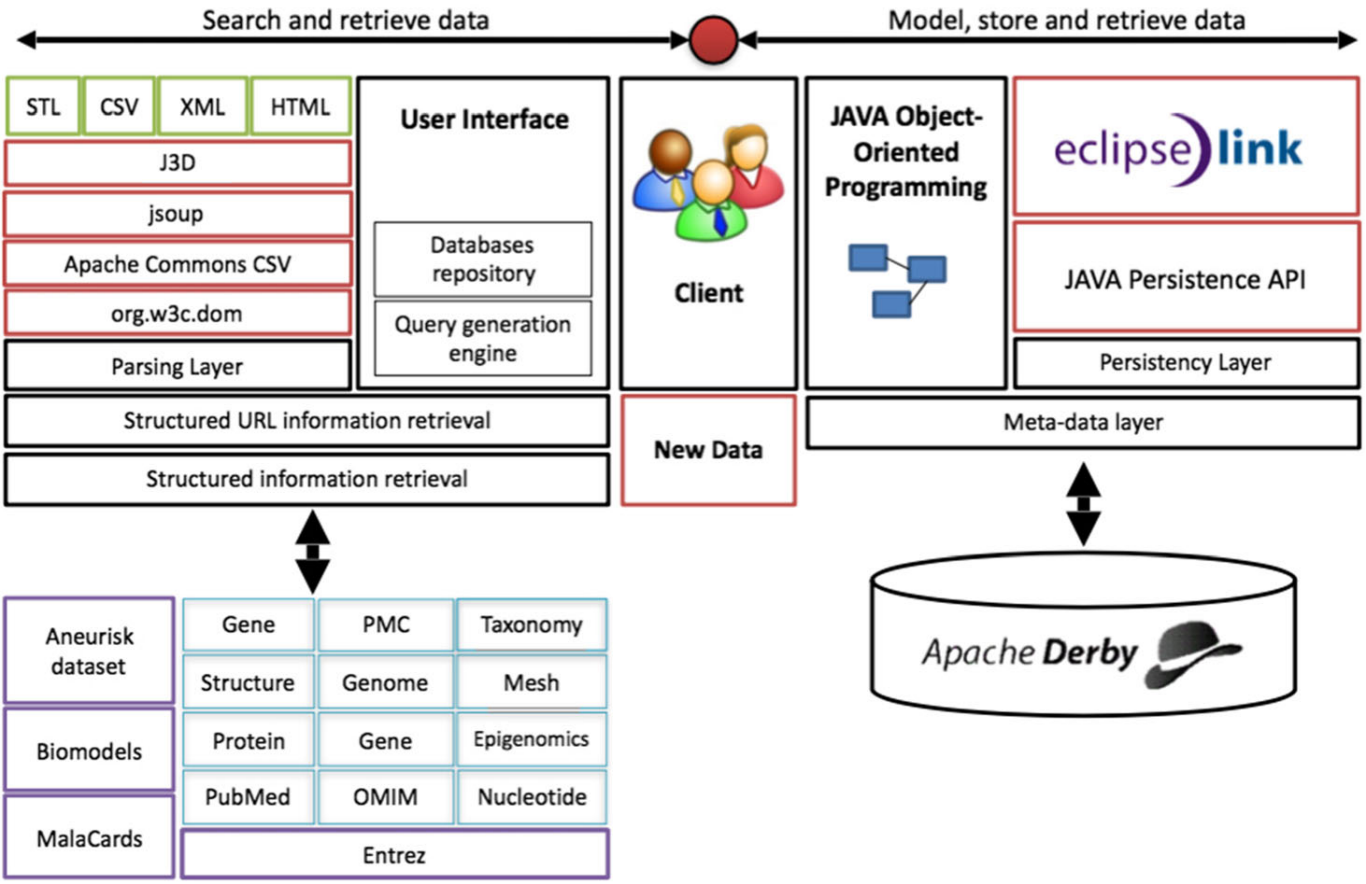
Implemented framework. Our implementation of the proposed framework utilizes Java as the development environment, EclipseLink as the JPA provider and Apache Derby as the database manager. Parsing layers were based on J3D, jsoup, Apache Commons and Org.w3c libraries. We implemented structured interfaces to various databases including MalaCards, Biomodels and several NCBI’s data sets

The proposed framework for the development of specialized databases can be implemented using different resources. Here, various open-source and free resources were utilized for implementation. Java was chosen as the development environment, EclipseLink as the JPA provider and Apache Derby as the database manager. Java was used to create interfaces to online databases such as MalaCards, Biomodels and NCBI’s databases. The structured information interface was used to derive data from local repositories such as Aneurisk, which was downloaded from the Aneurisk web dataset. By integrating these tools with a series of data parsers, a powerful framework for the curation of specialized databases is provided, which would incorporate new data and database-derived information into a user-defined database architecture. A schematic of the implementation is presented in Fig. 2.

The main data stream from established online databases was implemented using a structured URL interface. Databases often use a fixed URL syntax which translates a standard set of input parameters into the values necessary to search and retrieve requested data. For example, Entrez Programming Utilities provide a structured URL interface to the Entrez system, which currently includes 38 databases covering a variety of biomedical data, including nucleotide and protein sequences, gene records, three-dimensional molecular structures, and the biomedical literature [21]. A series of data processing tools were utilized to implement parsers for syntactic analysis of the retrieved data. The w3c.dom package provides the Document Object Model (DOM) interfaces, which were used as the API for XML processing. The Apache Commons’ libraries, the jsoup library and the org.j3d library of the Java 3D Community were utilized for CSV, html and STL parsing, respectively. An API with which the user can specify the required database from which she wants to search and retrieve data was developed. This API contains an expandable database repository and a simple query generation engine which can be used to search a specified database. The user uses Java OOP to encapsulate the retrieved data and integrate it with her own data. The mapping of Java objects and database tables is defined via persistence metadata, which is used by the JPA provider - EclipseLink - to execute database SQL-like operations for static and dynamic queries. EclipseLink defines the metadata via annotations in the Java class and with XML. The open source relational database Apache Derby - part of the Apache DB Project – was used as the database manager. Derby is written in java and is suitable for embedding due to its limited footprint and ease of use. Derby supports SQL data storing and querying in a client/server operation mode, commonly used by specialized databases.

The framework consists of five packages, each encapsulating a family of associated functionalities. The UML package diagram is shown in Additional file 1: Figure S1. The database package contains enumerated types which represent specific search fields for advanced search, types of retrieved information and available databases. The URL Interfaces package consists of a series of classes providing structured URL access to the different databases. The parsers package consists of classes for HTML, XML and CSV parsing. Each of those classes is generalized by model specific parsers as will be discussed below. The Persistency package consists of the persistent Agent class that can persist the model’s objects to memory. Finally, the model package consists of the model’s specific classes as will also be discussed below. Simplified UML views of the classes’ diagram of each of the packages are shown in Additional file 1: Figures S2–5.

The use of the system is quite simple. For example, a query for retrieving two articles according to their PMID’s from NCBI’s Pubmed database can be generated using:

**Table:** 

Query query = new Query(); query.setDatabase(DBType.PUBMED); query.addId(“23371018”); query.addId(“10227670”); query.setSearchType(SearchType.FETCH);	

The following structured URL is automatically generated by the framework as a result:

**Table:** 

Query URL: http://eutils.ncbi.nlm.nih.gov/entrez/eutils/efetch.fcgi?db=pubmed&id=23371018,10227670&retmode=xml	

More complicated queries can also be produced. For example, to retrieve publication ids for all articles published in the journal ‘Science’ in 2009, with the terms “breast” and “cancer”, the following query can be generated:

**Table:** 

Query query = new Query(); query.setDatabase(DBType.PUBMED); query.addTerm("breast"); query.addTerm("cancer"); query.addField(SearchFields.JOURNAL, "science"); query.addField(SearchFields.PUBLICATION_DATA, "2009" ); query.setSearchType(SearchType.SEARCH); List < String > results = Entrez.searchEntrez(query);	

As a result, the following structured URL is automatically generated by the framework:

**Table:** 

Query URL: http://eutils.ncbi.nlm.nih.gov/entrez/eutils/esearch.fcgi?db=pubmed&term=science[journal]+2009[pdat]+breast+AND+cancer&retmode=xml&rettype=uilist	

The created query can be sent to the Entrez utilities system for the retrieval of the corresponding articles using:

**Table:** 

Document xmlDocs = Entrez.callEntrez(query);	

The retrieved XML documents can then be sent for parsing for the generation of a linked list of ‘Article’ objects:

**Table:** 

PubmedParser parser = new PubmedParser(xmlDocs); parser.parse(); List<Article> articles = parser.getArticles();	

Each of the articles can now be connected to other objects. They can then be persisted to memory using:

**Table:** 

for (Article article: articles){ persistAgent.PersistArticle(article); }	

Now, the articles can be retrieved from the persistency agent by simply calling on its showArticles function. This function makes use of a simple SQL-like query:

**Table:** 

Query q = entityManager.createQuery (“**SELECT a FROM Article a**”); List<Article> articleList = q.getResultList();	

The retrieved articles can then be published on the command line to produce:

**Table:** 

--------------------------------------- ID: 23371018 TITLE: Non-dimensional analysis of retinal microaneurysms: critical threshold for treatment. AUTHOR: Ezra Elishai JOURNAL: Integrative biology : quantitative biosciences from nano to macro 5(3), 2013, DOI: 10.1039/c3ib20259c --------------------------------------- ID: 10227670 TITLE: Three dimensional analysis of microaneurysms in the human diabetic retina. AUTHOR: Moore J JOURNAL: Journal of anatomy 194 (Pt 1)(?), 1999, DOI: ? ---------------------------------------	

## Results

The proposed framework can be easily adopted for the curation of specialized databases. Here, the curation of a specialized database aimed to store and retrieve aneurysm-associated data is demonstrated. Aneurysms characterize important vascular pathologies, which depending on the aneurysm’s location and geometry could potentially cause blindness, stroke and death [22]. The query generating engine was used to retrieve tens of thousands of aneurysm related research articles from NCBI’s Pubmed and PMC databases. XML parser was utilized to analyze and store the retrieved data in a linked list of ‘Article’ objects; this list encapsulates each of the articles’ information. Abstracts are automatically downloaded and stored in the database for easy retrieval. The structured URL interface to the MalaCards database was used to retrieve hundreds of data records of associated human diseases such as Aortic aneurism and Cerebritis. The HTML parser was used to parse the information regarding the key characteristics of each disease, which were stored in a linked-list of ‘Disease’ objects.

For example, the following simple query for “aneurysm”:

**Table:** 

Query query = new Query(); query.setDatabase(DBType.MALA_CARDS); query.addTerm(“aneurysm”); Document results = MalaCards.callMalaCards(query);	

can be sent to the MalaCard parser to retrieve related diseases:

**Table:** 

MalaCardsParser parser = new MalaCardsParser(results, query); parser.parse(); List<Disease> diseases = parser.getDiseases();	

After persistency is performed (as was earlier described), the retrieved list of diseases can be published to produce (only the first 7 retrieved diseases are shown here):

**Table:** 

--------------------------------------- Name: Familial Thoracic Aortic Aneurysm and Dissection Link at MalaCards:/card/familial_thoracic_aortic_aneurysm_and_dissection?search=aneurysm --------------------------------------- Name: Coronary Aneurysm Link at MalaCards:/card/coronary_aneurysm?search=aneurysm --------------------------------------- Name: Angiopathy, Hereditary, with Nephropathy, Aneurysms, and Muscle Cramps Link at MalaCards:/card/angiopathy_hereditary_with_nephropathy_aneurysms_and_muscle_cramps?search=aneurysm --------------------------------------- Name: Aneurysmal Bone Cysts Link at MalaCards:/card/aneurysmal_bone_cysts?search=aneurysm --------------------------------------- Name: Intracranial Berry Aneurysm Link at MalaCards:/card/intracranial_berry_aneurysm?search=aneurysm --------------------------------------- Name: Cerebral Aneurysms Link at MalaCards:/card/cerebral_aneurysms?search=aneurysm --------------------------------------- Name: Loeys-Dietz Syndrome Link at MalaCards:/card/loeys_dietz_syndrome?search=aneurysm ---------------------------------------	

The platform was similarly used to download tens of related biological models such as the differentiation of endothelial cells, to download the related XML files, parse them, and encapsulate the data in a linked-list of ‘Model’ objects. Two of them are:

**Table:** 

--------------------------------------- Id: BIOMD0000000058 Description: The model reproduces the same amplitude antiphase calcium oscillations of coupled… --------------------------------------- Id: BIOMD0000000291 Description: adsorption of albumin-bilirubin complex to the surface of carbon pyropolymer… ---------------------------------------	

The structured information interface, the CSV parser and the STL loader were utilized to parse data from the Aneurisk repository, which contains clinical data and 3-dimensional models of hundreds of aneurisms. Examples of retrieved patients’ information are:

**Table:** 

--------------------------------------- Patient ID: C0004 SEX: F, AGE: 60 Aneurysm type: TER, location:ICA, status: U --------------------------------------- Patient ID: C0005 SEX: F, AGE: 26 Aneurysm type: LAT, location:ICA, status: R --------------------------------------- Patient ID: C0006 SEX: F, AGE: 45 Aneurysm type: LAT, location:ICA, status: U --------------------------------------- Patient ID: C0007 SEX: F, AGE: 44 Aneurysm type: LAT, location:ICA, status: U --------------------------------------- Patient ID: C0008 SEX: M, AGE: 68 Aneurysm type: TER, location:ACA, status: R ---------------------------------------	

**Fig. 3:**
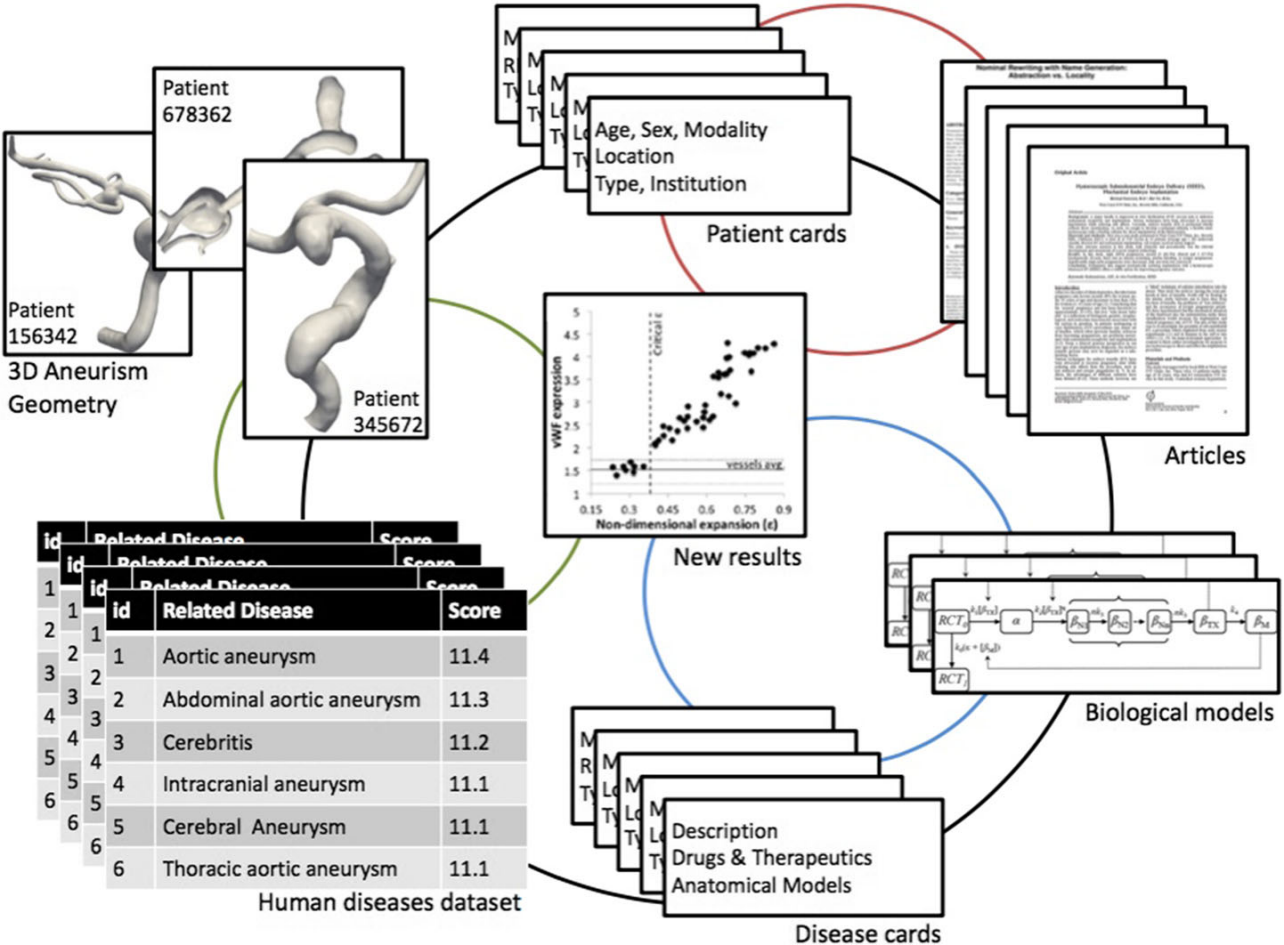
Construction of a specialized database. We have demonstrated generation of a specialized database for aneurysm-related vascular pathologies. This database contains 3 dimensional geometries of aneurysms, patients’ clinical information, articles, mathematical biological models, related diseases and our model of aneurysms’ risk of rapture

Aneurysms’ data was integrated with our previously published criteria of aneurysm risk of rapture [7]. A schematic of the database with its different associations is shown in Fig. 3. Simplified UML views of the models’ related classes are presented in Additional file 1: Figures S6–7.

The persisted database can now be inquired with SQL-like commands. For example, all relevant information regarding a patient – including articles, models and diseases – can be easily retrieved according to its identification number (in this case: CD0985674) using:

**Table:** 

Query q = entityManager.createQuery ("**SELECT p FROM Aneurysm p WHERE p.patientID = :patientID**"); q. setParameter(**"patientID", "CD0985674"**);	

## Discussion

In the last two decades a tremendous interest has developed in computational biology and bioinformatics, disciplines which have emerged from the intersection of biology and computer science. Practically, bioinformatics became a fertile new ground for programmers, who have gained access to an entirely new class of questions and challenges [23]. Commonly used software packages are the Bio*.org projects, such as BioRuby [24], BioPerl [25], BioJava [26] and BioPython [20], which have recently been assembled under the Open Bioinformatics Foundation. Each of these projects represents an international association of developers of open source code libraries for bioinformatics, genomics and life science research. However, these platforms are not oriented for the curation of databases. Sequence and non-bibliographic databases constitute the most important corner stone for research in computational biology and bioinformatics. While primary databases such as NCBI’s Nucleotide and Protein databases are of great importance to biological research, specialized databases which serve specific research communities are rapidly developing. During the last decade hundreds of specialized databases have been developed, each makes use of different frameworks and libraries’ sets. Although many database development environments exist, they often rely on a tabular structure, where the designer creates objects such as tables, columns, keys, indexes, relationships and constraints. While those basic entities are prevalent for simple data organization, they can rarely answer the needs of researchers who make use of a wide spectrum of data types, from sequencing data and microarray experiments to statistical models and simulations.

Here, a simple and unified open-source framework for the rapid development of specialized databases, based on user-defined objects and relations is proposed. These objects can be designed with the full arsenal of tools in OOP, giving the user maximum flexibility. It is important to note that the proposed framework aim to assist developers, which are capable of building object-oriented data models. After defining the data model (as it was exemplified in Additional file 1: Figure S7), developers can use the framework to load it with data from the supported web/local-based repositories, and persist/retrieve it from memory. Moreover, this framework can be rapidly extended or modified to support additional parsers and databases. The framework allows the user to concentrate on the biological models, the new data and the database architecture, rather than on concerns regarding data management and access to the different online and local datasets. This implementation is provided with a set of free, open source tools, to increase availability and to enable ease of use. The framework can be easily utilized to work with the variety of bioinformatics tools available via the open sourced BioJava project.

The most important aspect in the proposed work is the integration of the most relevant technologies to OO-based data-base design in a single framework. This is in clear contrast to BioPython/BioJava that emphasize utilization of algorithms for bioinformatics. While the proposed framework is focused on database design, BioPython/BioJava are focused on fundamental bioinformatics tasks ranging from sequence alignment and molecular structure prediction. Obviously, some aspects of the proposed framework such as interfacing web-based databases are congruent with BioPython/BioJava. However, the context of use is largely different. Moreover, by utilizing flexible structuring of URLs, the proposed framework also supports interfaces to databases such as MalaCards and BioModels. We note that another project – BioServices [27] - does indeed provides an interface to BioModels. Moreover, our framework provides an interface to Apache Derby – a strong ORM-based database manager. This is currently, to our knowledge is not embedded within BioPython/BioJava. We note that another python package named BioSQL [28], offers interface for SQL-relational data bases, in contrast to our framework, which is based on ORM.

The creation of a specialized database for aneurysm-associated data, and possible queries was demonstrated. A more substantial ‘data to knowledge’ utility of this database can be developed. For example, our predictive model of aneurysms’ risk of rapture uses a non-dimensional analysis of fluid dynamics to set a critical geometrical threshold of treatment [7]. Piccinelli and colleagues could derive geometrical measures of patients’ aneurysms’ geometries, which can be used with the prediction model to determine risk of rapture [29]. These patients’ geometries are incorporated as STL files in this database. Specialized database of aneurism-related data can be used to further investigate the links between the different aneurysm-associated diseases and the underlying biological models to advance knowledge in this field.

## Additional file

Additional file 1: Supplemntary information: framework's design and architecture. (DOCX 500 kb)
