# Supplementary material for: Rapid development of entity-based data models for bioinformatics with persistence object-oriented design and structured interfaces
Source: BioData Min. 2017 Mar 11;10:11. doi: 10.1186/s13040-017-0130-z (PMC5346198; doi:10.1186/s13040-017-0130-z)
Supplement: Additional file 1: — Supplemntary information: framework's design and architecture. (DOCX 500 kb) [file 13040_2017_130_MOESM1_ESM.docx]

**Additional file 1**

**Rapid development of entity-based data models for computational biology with persistence object-oriented design and structured interfaces**

Elishai Ezra

Department of Computer Science, Faculty of Engineering, Jerusalem College of Technology

**
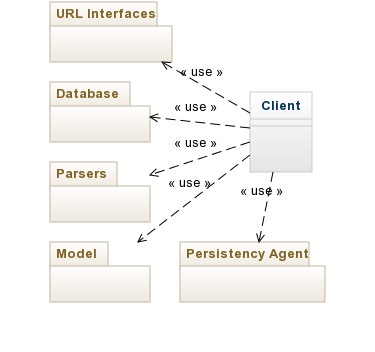
**

**Figure S1. *Packages view of the implanted framework***

Our framework consists of five packages: a database, URL Interfaces, parsers, a persistency agent and a model, each encapsulating a family of associated functionalities**.** The main class – client – can interact with each of the implemented functionalities for the curation of her database.


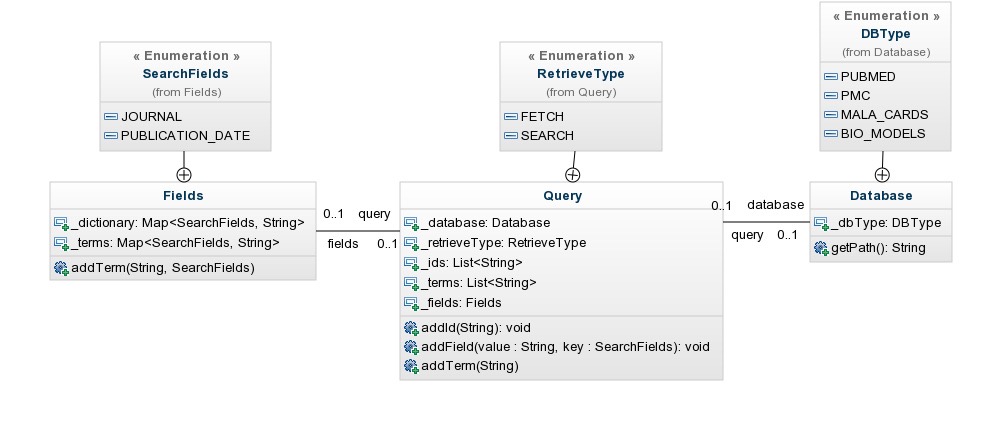


**Figure S2. *A simplified UML classes view of the Database package***

The database package contains enumerated classes which represent search fields for advanced searches, types of retrieved information and available databases. The class 'query' contains all relevant data for fetching or searching information. The class 'fields' provides a dictionary which can be used to search terms in pre-specified fields.


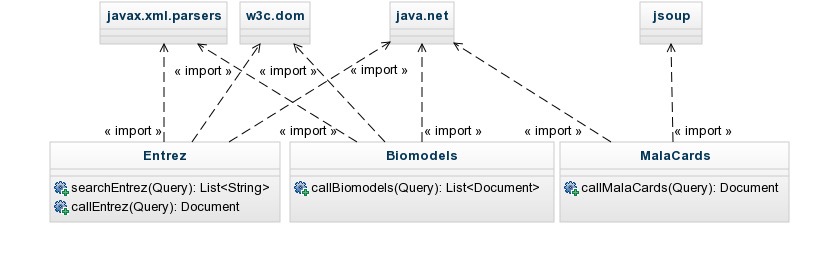


**Figure S3. *A simplified UML classes view of the URL interfaces package***

The URL Interfaces package consists of a series of classes which provide structured URL access to the different databases. All classes use java.net library to access the web. The Biomodels and Entrez interfaces use 'javax.xml' and 'org.w3c' external libraries to handle XML documents and MalaCards interface uses the jsoup library to handle HTML files.

**
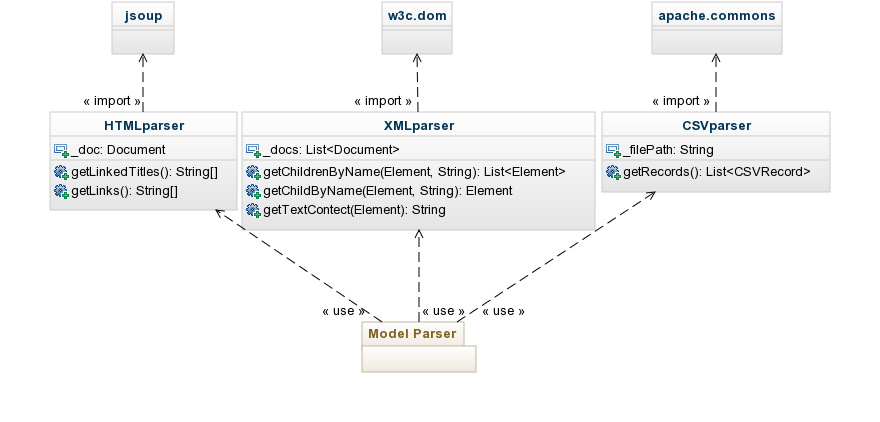
**

**Figure S4. *A simplified UML classes view of the parsers package***

The parsers package consists of classes for HTML, XML and CSV parsing using the 'jsoup', 'w3c' and 'apache.commons' external libraries respectively. Each of the aforementioned classes is generalized by the model's specific parser.

**
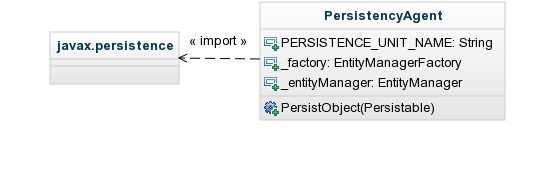
**

**Figure S5. *A simplified UML classes view of the persistency package***

The Persistency package consists of the persistentAgent class which loads the model's objects to memory using the 'javax.persistence' library.

**
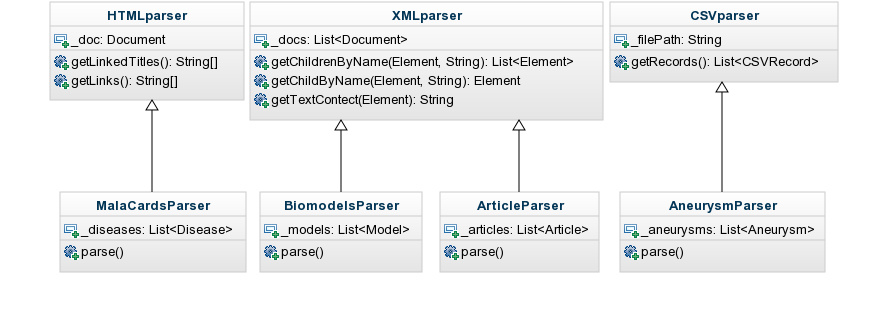
**

**Figure S6. *A simplified UML classes view of the model parsers package***

A parser class is written for each of the model's classes. The parsers for Biomodels and articles generalize the XMLparser class, the aneurysm parser generalizes the CSV parser and the MalaCards parser generalizes the HTML parser.

**
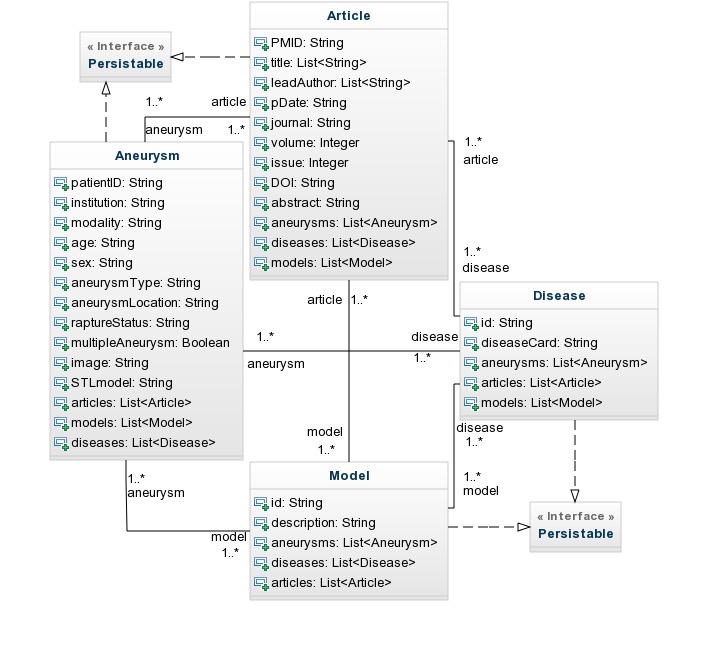
**

**Figure S7.
A simplified UML classes view of the models package**

The data model consists of four classes, encapsulating a disease, an aneurysm, an article and a model. All of the classes are connected as attributes.

# 
